# Editorial: Case reports in pediatric cardiology 2022

**DOI:** 10.3389/fped.2023.1298526

**Published:** 2023-10-13

**Authors:** Alvise Guariento, Francesco Bertelli, Vladimiro L. Vida

**Affiliations:** Pediatric Cardiac Surgery, Department of Cardiac, Thoracic, Vascular Sciences and Public Health, University of Padua, Padua, Italy

**Keywords:** pediatric cardiology, congenital heart disease, editorial, research topic, congenital cardiac surgery

**Editorial on the Research Topic** Case reports in pediatric cardiology 2022

Pediatric cardiology stands as a distinctive subset within the field of cardiology, which typically brings to mind cardiovascular issues prevalent among older individuals. Yet, the truth is that the heart is an incredibly resilient organ, capable of allowing years of relatively symptom-free survival even when significantly damaged. Regrettably, children can and do encounter cardiovascular problems, although their occurrence remains considerably lower compared to the adult population. This reality carries numerous implications. Firstly, due to social and economic factors, scientific and clinical research predominantly focuses on diseases with higher prevalence. Consequently, pediatric cardiology remains, and likely always will be, an area of study that receives less attention. Secondly, as children grow and naturally outgrow surgically implanted prosthetic devices or tissues, this presents unique challenges in their treatment.

One of the most captivating facets of pediatric cardiology lies in its close connection to the intriguing realm of congenital heart disease (CHD). This umbrella term encompasses structural or functional abnormalities of the heart or major blood vessels that originate before birth. While most CHD occur independently of other diseases, severe and complex forms often coexist with additional medical conditions.

It is essential to acknowledge how CHD can sometimes manifest in unconventional ways, resembling conditions such as ischemic heart disease. A noteworthy example is a case reported by Fallah et al. involving a 2-year-old child with left main coronary artery atresia. This child exhibited classical features of ischemic heart failure, including failure to thrive, a significantly dilated left ventricle, an ejection fraction of 20%–25%, and severe functional mitral regurgitation. The diagnosis was initially suspected due to cardiac magnetic resonance findings, which indicated ischemic heart disease through extensive scarring of the left ventricle.

Another intriguing case is presented by Wu et al. involving a term female newborn who presented with congestive heart failure and a grade 4/6 continuous heart murmur in the left upper sternal border. This child was diagnosed with a giant fistula between the right coronary artery and the right ventricle, with an orifice of approximately 2 mm. What makes this case remarkable is the successful percutaneous closure of the fistula using an Amplatzer Duct Occluder II, originally designed for patent ductus arteriosus (PDA) closure. This was possible due to the severe aneurysmatic dilatation of the proximal right coronary artery, which could accommodate both the guidewire and the device itself.

In some instances, coronary artery anomalies can be challenging to diagnose, as demonstrated in the case reported by Hu et al. In this case, a seven-year-old male child experienced sudden, sharp chest pain and syncope during high-intensity exercise. While cardiac ultrasound initially showed a normal right coronary artery, the left coronary artery exhibited non-continuous blood flow, raising suspicion of a coronary artery anomaly. Subsequent electrocardiogram (EKG)-gated computed tomography (CT) angiography revealed an abnormal origin of the left coronary artery from the upper portion of the non-coronary sinus, with a lengthy stenotic intra-mural course. This case highlights the importance of advanced imaging when there is a high clinical suspicion of a coronary artery anomaly.

Anomalous aortic origin of a coronary artery (AAOCA) is a prevalent congenital coronary artery anomaly that can lead to syncope in children. In fact, AAOCA is the second most common cause of sudden cardiac death in young athletes (1), as reported by Gao et al. in a case series of pediatric patients admitted to their hospital due to syncope. Namely, the stories of a total of eight patients with an average age of 12.5 years were described. However, not all cardiovascular problems in children are congenital; various other causes, including trauma, are possible, as documented by Ai et al. Their case involved an eight-month-old female child who underwent elective corrective surgery for Tetralogy of Fallot but experienced a life-threatening right coronary artery rupture as a result of cardiopulmonary resuscitation (CPR). This case highlights that coronary artery rupture can occur as a complication of CPR, an occurrence documented in limited literature.

While pediatric cardiologists predominantly focus on CHD, acquired cardiovascular issues commonly seen in adults can also affect previously healthy children. This includes both common conditions like myocarditis and acute myocardial ischemia, as well as exceptionally rare conditions such as pulmonary artery dissection (PAD). Most cases of PAD arise due to medial degeneration, characterized by the fragmentation of elastic fibers and the widespread enlargement of pulmonary arterial branches, typically resulting from chronic pulmonary hypertension. However, Ren et al. reported two infant patients with PAD who lacked pulmonary hypertension and underlying medical conditions. Notably, these patients presented with recurrent pneumonias, lacking the typical symptoms of chest pain and hemoptysis seen in adults.

While many CHDs lack a clearly defined genetic basis, some genetic diseases directly or indirectly affect cardiovascular and pulmonary physiology. Lin et al. reported the case of a newborn boy who suffered from severe dyspnea, extreme anemia, skin pallor, and hypoxemia due to severe pulmonary hypertension. Whole-exome sequencing revealed a novel compound heterozygous mutation in the gene encoding the Pyruvate Kinase enzyme, leading to a diagnosis of pyruvate kinase deficiency (PKD). Treatment of the underlying condition also resolved the pulmonary hypertension, highlighting the importance of precise diagnosis, even when a genetic component may not be immediately apparent.

Liu et al. described the case of a 21-year-old man diagnosed with arrhythmic-dilated cardiomyopathy secondary to Duchenne muscular dystrophy, primarily treated with steroids. This case underscores the significance of cardiological follow-up in all patients at risk of developing heart problems, even if such cases are rare. Another example comes from Feng et al. who reported the case of a 17-year-old girl with Axenfeld-Rieger syndrome (ARS), an autosomal dominant disorder linked to disruption of the development of neural crest cells. While cardiac defects associated with ARS have been reported, this patient presented with a range of cardiac malformations not previously described. This suggests the need for echocardiography in patients with characteristic clinical manifestations of ARS or specific genetic alterations.

Generalized arterial calcification of infancy (GACI) is an autosomal recessive condition characterized by extensive calcification and intimal proliferation of the large and medium arteries, including the aorta, coronary arteries, and renal arteries (2). This leads to vascular stenosis and a range of complications, such as severe systemic hypertension and heart failure. Făgărășan et al. presented a case of successful surgical treatment of severe aortic arch obstruction caused by calcified plaques mimicking severe coarctation of the aorta. Additionally, Lu et al. reported a case of GACI in an 8-month-old boy who presented with hypertension, hypertrophic cardiomyopathy, and heart failure, ultimately leading to his demise before bisphosphonate treatment could be initiated.

Mitochondrial diseases (MDs) are exceedingly rare (3), characterized by oxidative phosphorylation dysfunction due to nuclear and/or mitochondrial DNA variations (4). Wang et al. described the case of an 8-month-old male with MD, initially presenting with severe lactic acidemia and respiratory distress, along with echocardiographic features suggesting hypertrophic cardiomyopathy. The importance of this case lies in the correlation between MD and cardiac manifestations, highlighting the need for a comprehensive investigation in such patients.

Chromosomal defects, particularly Turner syndrome, are strongly associated with CHDs. Lin et al. reported a case involving a patient with Turner syndrome who had severe aortic coarctation. They successfully deployed a Cheatam-Platinum stent to address this condition, offering an alternative treatment method.

The integrity and configuration of vascular stents can easily be compromised with aggressive manipulation, making percutaneous interventions more challenging and technically intricate. Currently, there are no established protocols for reclaiming embolized strutted stents through percutaneous means. Prakoso et al. recounted their experience in retrieving a strutted stent from the abdominal inferior vena cava of a three-month-old boy scheduled for femoral transvenous ductal stenting (DS). Due to complex angulation, inserting the stent into the PDA proved technically unfeasible. However, they successfully recaptured the stent using a gooseneck snare through a right atrial appendage (RAA) hybrid access, all without the need for cardiopulmonary bypass support.

Patients with Fontan circulation present distinct challenges related to their cardiopulmonary function. As a result, ongoing research seeks to determine whether COVID-19 poses an increased risk to this specific population. Wen et al. detailed the case of a nine-year-old male child who underwent Fontan palliation and later contracted COVID-19 during the pandemic. This case prompted investigations into the unique therapeutic needs of Fontan patients. While complications were not uncommon in this population, thrombotic complications were the most frequent. However, these complications did not appear to be specific to Fontan circulation, and most patients ultimately improved and fully recovered. Notably, worse physiological conditions like cyanosis and pulmonary hypertension were associated with higher mortality rates.

At the outset of the COVID-19 outbreak, children were minimally affected, accounting for only 1.7% of cases and often presenting as asymptomatic carriers. Nevertheless, as the pandemic progressed, a growing number of children exposed to the virus developed Multisystem Inflammatory Syndrome in Children (MIS-C). Di Filippo et al. provided an extensive case series shedding light on cardiac manifestations observed during COVID-19 in children and highlighting the significance of elevated troponin levels. Among their cases, 13.6% exhibited various forms of cardiac involvement, and 9.6% showed elevated troponin levels. Given the ongoing COVID-19 pandemic that has persisted in recent years and the widespread vaccination efforts, it is unsurprising that adverse reactions to vaccination, even in children, have been reported. The use of COVID-19 vaccines is now recommended for the pediatric population. Lu et al. presented a noteworthy case series detailing their experience with adverse reactions to the hepatitis B vaccine, aiming to provide insights into the general mechanisms underlying vaccine adverse reactions. Of the adverse events documented, three were cases of myocarditis, two were meningitis, and two were interstitial pneumonia. A similar case of adverse reaction to an RNA COVID-19 vaccine in a pediatric patient was described by Han et al. They presented the medical history of a 17-year-old female patient who experienced chest pain and syncope following her initial dose of the messenger RNA COVID-19 vaccine. Subsequent cardiac magnetic resonance imaging confirmed the diagnosis of myocarditis based on established criteria.

Dilated cardiomyopathy (DCM) stands as one of the primary causes of heart failure in children, with heart failure often being the initial presentation, though clinical manifestations can vary. Wang et al. reported the first documented case of marked right atrial (RA) enlargement as the initial presentation of DCM. Genetic analysis revealed a heterozygous mutation associated with cardiomyopathies. Further sequencing identified the same variant in Pkp2 in the patient's asymptomatic mother, whose echocardiography showed an enlarged left atrium (LA) and left ventricle (LV), mild to moderate mitral regurgitation, and a reduced left ventricular ejection fraction (LVEF) of 48%. Thus, she was also diagnosed with DCM, establishing a familial DCM diagnosis based on the patient's and mother's features.

Zhang et al. presented another unique case: the first instance of fetal non-compaction cardiomyopathy occurring simultaneously in both ventricles, coupled with the identification of a mutation in the calmodulin gene (CALM2). Prenatal echocardiography initially detected biventricular non-compaction cardiomyopathy alongside sinus bradycardia. Following the termination of the pregnancy, autopsy and histopathological examination confirmed the diagnosis of fetal biventricular non-compaction cardiomyopathy.

Torsades de pointes (Tdp) represents a life-threatening ventricular tachyarrhythmia characterized by a constantly shifting QRS complex morphology, twisting the electrical axis around the isoelectric line. Wang et al. conducted a study to document the diagnosis and management of a rare case involving frequent Tdp in a child with a novel genetic mutation. The patient was successfully treated with a cardioverter-defibrillator (ICD) implantation and optimization of antiarrhythmic therapy.

Atrial tachycardia (AT) originating from the atrial appendage (AA) is clinically characterized by palpitations, chest discomfort, dyspnea, and other nonspecific symptoms. In children, AT originating from the AA accounts for approximately 30%–50% of AT cases, a higher incidence than in adults (5). Liu et al. reported three cases of AT originating from the AA, treated with a combination of three-dimensional electroanatomic mapping and ablation, and surgical atrial appendage resection performed in conjunction with cardiac surgery. Atrial fibrillation (AF) is an uncommon occurrence among children, especially in the absence of underlying congenital heart disease (6). Pediatric epidemiological data on AF are limited, often relying on findings from studies conducted in the adult population. When AF is diagnosed in a young patient with a structurally normal heart, a comprehensive investigation into its underlying cause becomes essential. Hubrechts et al. presented a rare and potentially life-threatening origin of AF: intrathoracic non-Hodgkin lymphoma with cardiac involvement, as revealed by cardiac magnetic resonance imaging (CMR). This represents the first documented pediatric case attributing new-onset AF to neoplastic infiltration of the left atrial wall.

Catecholamine-induced cardiomyopathy is a rare and challenging complication associated with phaeochromocytoma-paraganglioma, more commonly observed in pheochromocytoma but less common in neuroblastoma (NB). Xu et al. presented the case of a 5-year-old girl with NB who developed catecholamine cardiomyopathy, specifically hypertrophic cardiomyopathy (HCM), leading to ventricular hypertrophy, hypertension, and heart failure. Surgical removal of the tumor resulted in the normalization of blood pressure and urinary catecholamine levels. A 7-month follow-up revealed the resolution of ventricular hypertrophy and the restoration of normal ventricular function.

Restrictive cardiomyopathy (RCM) represents the least common phenotype among pediatric heart muscle diseases, accounting for approximately 5% of all diagnosed cardiomyopathies, and is associated with a poor prognosis in children (7). Ji et al. reported a case of RCM that initially manifested with ventricular fibrillation in a 7-year-old boy who was successfully resuscitated by an automated external defibrillator (AED) outside the hospital. At present, the boy is being treated with oral diuretics and metoprolol tartrate tablets, as his parents declined an ICD implantation, and he is undergoing outpatient follow-up.

Heart–lung transplantation (HLT) remains the sole viable treatment for certain advanced cardiopulmonary diseases. However, the scarcity of donors, the necessity for intricate surgical coordination, and the demanding post-operative care limit the number of such procedures performed in children worldwide. Post-transplantation challenges persist, including rejection, infections, renal issues, tumors, and other complications that can adversely impact patients’ quality of life. Zhuang et al. documented a case of cerebral aspergillosis in a 10-year-old child that developed three months after HLT. Fortunately, the patient responded well to treatment, and there were no recurrences of the disease during the 3-year follow-up period.

Idiopathic pulmonary arterial hypertension (PAH) is a rare and progressively debilitating condition affecting the pulmonary arteries. Epoprostenol, a synthetic prostaglandin analog, stands out as the most potent pharmaceutical option for treating PAH. Chida-Nagai et al. shared their experience with an adolescent female patient who successfully transitioned from continuous intravenous epoprostenol therapy to gradual oral selexipag administration over an extended period. Encouragingly, this shift proved effective, suggesting that oral selexipag can offer comparable efficacy to epoprostenol, especially for managing PAH in young patients.

Lian et al. presented a case involving an eight-year-old child with an exceedingly rare combination of right aortic arch, right patent ductus arteriosus (PDA), and right tracheal bronchus, a condition known since birth. Interestingly, the patient later developed symptoms of airway compression, prompting surgical intervention involving the ligation and division of the PDA through a standard midline sternotomy. This case is remarkable not only for its unprecedented combination but also because the patient remained asymptomatic for many years despite the congenital anomaly being known since birth.

Hypoplastic left heart complex (HLHC), which also encompasses Shone's syndrome, constitutes a rare congenital heart disease (CHD) characterized by severe obstructive lesions in the left-sided inflow and outflow tracts (8). While supramitral fibroelastic membranes contributing to mitral valve (MV) obstruction are common in this disease entity, left ventricular endocardial fibroelastosis (EFE) has not typically been considered a major factor in Shone's variant HLHC. Diaz-Gil et al. provided the first description of active clinical manifestation of EFE and remodeling of the endocardium through endothelial-to-mesenchymal transformation (EndMT) in an adolescent with Shone's variant HLHC and a genetic heterozygous ABL1 variant. This case highlights the need for novel therapeutic approaches for EFE, potentially focusing on molecular factors influenced by intrinsic and extrinsic stimuli of EndMT.

Surgery is the standard approach to correct ventricular septal defects (VSDs), especially in complex cases involving individuals with pulmonary hypertension and multiple defects. In recent years, transcatheter percutaneous closure has gained favor, particularly for muscular VSDs located in challenging surgical sites. However, repairing multiple defects often involves using multiple devices and typically relies on fluoroscopy guidance (9). Siagian et al. detailed their experience with the closure of multiple VSDs using a single device and a zero-fluoroscopy technique in a 7-year-old patient who had experienced shortness of breath for a year prior to admission. They employed a jugular vein approach to successfully perform percutaneous transcatheter VSD closure. Remarkably, 1.5 years after the procedure, any visible signs of pulmonary hypertension had resolved, leading to the discontinuation of pulmonary artery dilator treatment.

In conclusion, it is crucial to raise awareness of pediatric cardiology among healthcare professionals, and the objective of these case series is precisely that. Through the sharing of a collection of rare case reports, we aimed to foster an understanding of the various conditions’ potential manifestations and their corresponding treatments. This knowledge can significantly enhance clinical practices, diagnostics, and therapeutic interventions.
